# The efficacy and acceptability of exposure therapy for the treatment of post-traumatic stress disorder in children and adolescents: a systematic review and meta-analysis

**DOI:** 10.1186/s12888-022-03867-6

**Published:** 2022-04-12

**Authors:** Tengyue Huang, Haomiao Li, Shiyu Tan, Siyu Xie, Qisheng Cheng, Yajie Xiang, Xinyu Zhou

**Affiliations:** 1 Chongqing Medical University, Chongqing, China; 2 Department of Psychiatry, The First Affiliated Hospital of Chongqing Medical University, Chongqing, China; 3 Department of Neurology, The First Affiliated Hospital of Chongqing Medical University, Chongqing, China

**Keywords:** Exposure therapy, Post-traumatic stress disorder, Children, Adolescents, Meta-analysis

## Abstract

**Background:**

Posttraumatic stress disorder (PTSD) is common among children and adolescents who have experienced traumatic events. Exposure therapy (ET) has been shown to be effective in treating PTSD in adults. However, its efficacy remains uncertain in children and adolescents.

**Aims:**

To evaluate the efficacy and acceptability of ET in children and adolescents with PTSD.

**Method:**

We searched PubMed, EMBASE, Cochrane, Web of Science, PsycINFO, CINAHL, ProQuest, LILACS, and international trial registries for randomized controlled trials (RCTs) assessed ET in children and adolescents (aged ≤18 years) with PTSD up to August 31, 2020. The primary outcomes were efficacy (the endpoint score from PTSD symptom severity rating scales) and acceptability (all-cause discontinuation), secondary outcomes included efficacy at follow-up (score from PTSD scales at the longest point of follow-up), depressive symptoms (end-point score on depressive symptom severity rating scales) and quality of life/social functioning (end-point score on quality of life/social functioning rating scales). This study was registered with PROSPERO (CRD42020150859).

**Result:**

A total of 6 RCTs (278 patients) were included. The results showed that ET was statistically more efficacious than control groups (standardized mean differences [SMD]: − 0.47, 95% confidence interval [CI]: − 0.91 to − 0.03). In subgroup analysis, exposure therapy was more efficacious for patients with single type of trauma (SMD: − 1.04, 95%CI: − 1.43 to − 0.65). Patients with an average age of 14 years and older, ET was more effective than the control groups (SMD: − 1.04, 95%CI: − 1.43 to − 0.65), and the intervention using prolonged exposure therapy (PE) (SMD: − 1.04, 95%CI: − 1.43 to − 0.65) was superior than control groups. Results for secondary outcomes of efficacy at follow-up (SMD: − 0.64, 95%CI: − 1.17 to − 0.10) and depressive symptoms (SMD: − 0.58, 95%CI: − 0.93 to − 0.22) were similar to the previous findings for efficacy outcome. No statistically significant effects for acceptability and quality of life/social functioning were found.

**Conclusion:**

ET showed superiority in efficacy at post-treatment/follow-up and depressive symptoms improvement in children and adolescents with PTSD. Patients with single type of trauma may benefit more from ET. And ET is more effective in patients 14 years or older. Moreover, PE could be a better choice.

## Background

Post-traumatic stress disorder (PTSD) is the most common and typical continuous severe psychological disorder in individuals after exposure to an unusual threatening or catastrophic event [1–4]. Children who experience traumatic events may develop PTSD at higher rates than adults [5, 6]. It is reported that the overall rate of PTSD in trauma-exposed children and adolescents was 15.9% [7]. If PTSD is not treated, it may lead to additional psychiatric disorders such as depression, anxiety disorders, with functional impairment during both childhood and adulthood [8]. In many cases, PTSD can turn into a chronic disease, leading to considerable disease burdens and social and occupational disorders, huge economic and social costs and increased suicide risk [9, 10].

The psychotherapy is recommended by several clinical guidelines as the initial treatment of PTSD in children and adolescents [11–14]. In order to adequately process traumatic memories and ultimately eliminate fear, patients must reactivate their unwanted memories, and safety ingredients should be implanted [15]. Exposure therapy (ET), for traumatic memories, aims to deal with information including trauma situation and related emotions, thoughts and behaviors. ET has demonstrated its efficacy in the treatment of phobias, anxiety and PTSD in adults [16]. There are many kinds of exposure therapy available now, including prolonged exposure (PE), narrative exposure therapy (NET), kid narrative exposure therapy (KIDNET), etc. PE is a specific exposure-based type of cognitive behavior treatment for PTSD, and it is widely accepted in adults [17], by safe confrontation with thoughts, memories, places, activities and the people that have been avoided since a traumatic event occurred [18]. NET is a manualized, short-term, individual intervention program for the treatment of PTSD, based on CBT principles [19]. NET has been adapted for the use with traumatized children and adolescents in a version called KIDNET [20].

However, the gradual promotion of PTSD exposure therapy has caused some controversies in the treatment of PTSD in children and adolescents, which was mainly related to ethical aspects and safety issues [21]. A high drop-out rate of exposure therapy was reported because of the too low or too high patient engagement in the recalled memories during the treatment [22, 23]. Evidence from studies in the clinical settings among children was limited [24]. Therefore, we performed a meta-analysis to systematically evaluate the efficacy and acceptability of exposure therapy in the treatment of PTSD in children and adolescents.

## Method

The protocol of this meta-analysis has been registered in the PROSPERO database (CRD42020150859). The data that support the findings of this study is publicly available in Mendeley at 10.17632/d6m4xhwtyw.3. We defined the main structured research question describing the Population, Intervention, Comparison, Outcome, and Study design (PICOS) in accordance with the recommendations by the Preferred Reporting Items for Systematic Reviews and Meta-analysis (PRISMA) groups [25].

### Search strategy and selection criteria

We searched PubMed, EMBASE, Cochrane, Web of Science, PsycINFO, CINAHL, ProQuest Dissertations, LILACS, international trials registers (such as World Health Organization trials portal, ClinicalTrials. gov and Australian New Zealand Clinical Trials Registry) including published and unpublished trials, from the date of database inception to August 31, 2020. We put no restrictions on language. We searched with different combinations of the following keywords: Condition = (posttrauma* OR post-trauma* OR post trauma* OR trauma* OR PTSD OR Post-traumatic stress symptoms OR PTSS OR acute stress disorder* OR peritrauma* OR peri-trauma* OR avoidant disorder* OR combat disorder* OR war neurosis OR Schreckneurose OR fright neuroses OR shell shock OR sex*-abus* OR sex* abus* OR terror* OR war OR conflict* OR violen* OR acciden* OR shoot* OR disaster* OR earthquake OR tornado OR flood OR tsunami* OR hurricane* OR fire OR maltreat* OR crash* OR death OR grief) AND Intervention = (psychother* OR psychological OR cognitive-behavio* OR cognitive-behavio* OR behavio* OR cogniti* OR CBT OR exposure therapy OR exposure treatment OR exposure-based behavior therapy OR exposure-based OR exposure*).

Our inclusion criteria were as follows: (i) randomized controlled trials (RCTs); (ii) participants were children and adolescents (aged ≤18 years) who met the criteria for PTSD diagnosis: a. for patients with complete PTSD, according to the standardized diagnosis based on the international classification (the Diagnostic and Statistical Manual of Mental Disorders [DSM] [26–30], the International Classification of Diseases [ICD] [31, 32] or validated scales for PTSD based on DSM/ICD criteria [33–36]); b. patients with subclinical PTSD, defined as those who have experienced psychological trauma, present with at least one of the four symptom groups described in DSM-5 and reported some subsequent symptoms of PTSD, including re-experiencing, avoidance, overreaction, and negative cognitive and emotional changes; c. patients with clinically significant symptoms of PTSD, that was, the score of the patient scale was higher than the effective threshold of the PTSD rating scale; (iii) the intervention was exposure therapy, including PE, NET, KIDNET, etc.; (iv) there were more than ten participants per study; (v) the interventions of the control group were active control groups (ACG), treatment as usual (TAU), and waiting list (WL). The ACG could include supportive unstructured psychotherapy, nondirective supportive treatment and child-centered therapy.

The initial screening was based on the title and abstract by two researchers (T.H and L.H) independently. Publications were excluded from the search results if they did not meet the aforementioned inclusion criteria. Any disagreement during the process was resolved by discussing with senior reviewing authors (Y.X and X.Z). We contacted the corresponding authors in request for the data missing from the publication that was necessary for conducting the analysis. If the authors did not respond with the sufficient data to perform the meta-analysis or did not respond, the studies were excluded.

### Data extraction

Two independent reviewers (T.H and L.H) extracted the relevant parameters from the original paper, including the titles of the studies, patient characteristics (including type of trauma, diagnostic criteria for PTSD, severity of PTSD symptoms, the sample size, mean age and gender of participants), intervention details (including type of interventions, number of sessions, treatment duration, follow-up duration). If there was a disagreement between two reviewers, we resolved the disagreement by discussing with the senior review authors (Y.X and X.Z).

### Quality assessment

Two reviewers (S.T and S.X) assessed the methodological quality of the included studies independently. According to the Cochrane Collaboration’s tool V.2.0, the risk of bias was rated as ‘low risk’, ‘high risk’ or ‘some concerns’ in the following domains: (1) bias arising from the randomization process (Systematic differences in baseline characteristics of the comparison groups); (2) bias due to deviations from intended interventions (Systematic differences in care, exposure factors, etc. between groups, other than the intervention of interest); (3) bias due to missing outcome data (Systematic differences due to dropout of cases between groups); (4) bias in measurement of the outcome (Systematic differences in the measurement of outcomes between groups); (5) bias in selection of the reported result (Systematic differences between reported and unreported results). The disagreement between two reviewers was resolved by discussing with the senior review authors (Y.X and X.Z).

### Outcomes

The primary outcomes were the efficacy (as measured by the endpoint score from PTSD symptom severity rating scales completed by children, parents or clinicians) and acceptability (as the percentage of people who had dropped out from the study for any reason) of post-treatment. Assessment methods such as the Child PTSD Symptom Scale-Interview (CPSS-I), the Clinician-Administered PTSD Scale (CAPs) were used to measure the curative effect of complete exposure therapy for the treatment of children and adolescents with PTSD. If more than one scale was reported in a trial, we chose the scale with the highest ranking according to a hierarchy based on psychometric properties and appropriateness, which was defined in our previous registered protocol. If the trial had different raters of the assessment of PTSD symptom severity rating scale, self-report was prefered [37]. Secondary outcomes included efficacy at follow-up (measured by the score from PTSD scales at the longest point of follow-up up to 12 months), depressive symptoms (measured by the end-point score on depressive symptom severity rating scales), quality of life and functional improvement (QoL/functioning) (measured by the end-point score on QoL/functioning rating scales).

### Statistical analysis

Analyses were conducted by using the Review Manager, Version 5.3 and Stata 16.0. Continuous variables were estimated by pooled standardized mean differences (SMD, hedge’s g) and 95% confidence intervals (CIs). We weighted the within-study and between-study variance according to the size of the sample size of each independent study, taking into account the sampling error of the study and the error of the true effect size [38]. The binary variables were estimated by pooled odds ratios (ORs) and 95% CIs. The significance of the pooled SMDs or ORs was estimated by Z test (*P* < 0.05 was considered statistically significant). The I^2^-based Q statistic test was performed to evaluate variations due to heterogeneity rather than chance. A random-effects or fixed effects methods model was used to calculate the pooled effect estimates in the presence (P < 0.05) or absence (*P* ≥ 0.05) of heterogeneity. We assigned adjectives of low, moderate, and high to I^2^ values of 25, 50, and 75% [39].

Considering the possibility that effectiveness may differ according to different parameters, we conducted various subgroup analyses of the parameters as following: (1) single type of trauma vs. multiple types of trauma; (2) PE vs. NET vs. KIDNET; (3) treatment duration ≥12 weeks vs. treatment duration < 12 weeks [40]; (4) mean age < 14 years vs. mean age ≥ 14 years. We also performed sensitivity analyses by omitting RCTs published in a significantly different year than the others, or RCTs with non-blinding assessment, or RCTs with a small sample size. All tests were two-sided, and statistical significance was defined as a probability *P* value of < 0.05.

## Results

### Study characteristics

**Fig. 1:**
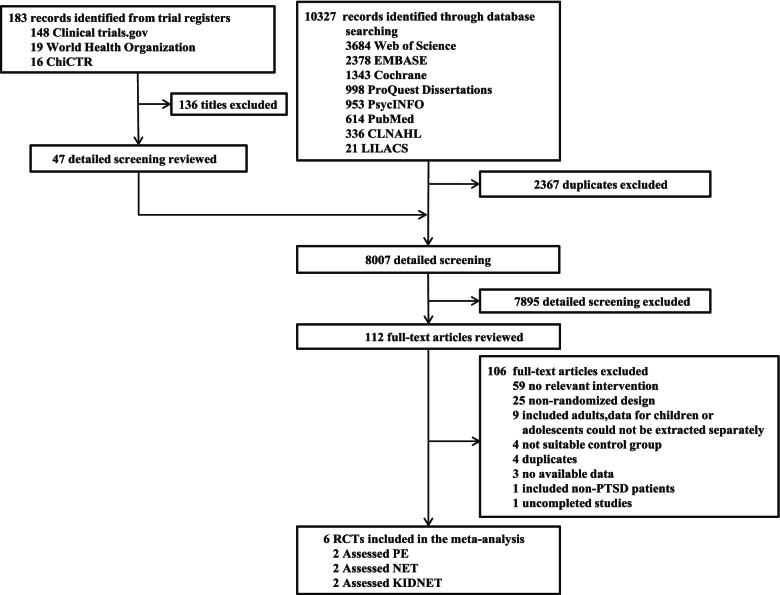
Flow chart of study selection. Abbreviations: PE = Prolonged Exposure Therapy, NET = Narrative Exposure Therapy, KIDNET = Narrative Exposure Therapy for children

Through searching the databases and international trials registers mentioned above, 10,510 citations were identified and 112 potentially eligible articles were reviewed in full text. In total, 6 clinical trials were included in the present study. The flow diagram was shown in Fig. 1. Six randomized controlled trials (*n* = 278) comparing ET (*n* = 145) with control conditions (*n* = 133) were included, the clinical and methodological characteristics of included trials were summarized in Table 1. The mean age was 13.84 years old (SD = 2.33) and 69.1% were females. 2 RCTs [41, 45] (33.33%) were conducted in Sri Lanka (*n* = 78), and four (66.67%) from other countries (Germany, Finland, South Africa, etc.). The mean study sample size was 46, ranging from 26 to 63 patients. Trauma types included trauma from the war [45] (n = 78), sexually abused [42] (*n* = 61), mixed [46] (*n* = 123) and others.

**Table 1:** Randomized controlled trials included in the systematic review and meta-analysis

Trial	Type of trauma	Diagnostic criteria	Treatments (number of sessions)	No. randomised	Age range and mean age (years)	Proportion of female (%)	Recruitment	Baseline severity scale	Mean baseline severity (SD)	Treatment Duration, (w)	Follow-up Duration, (m)
Catani 2009, [41]	War and Tsunami	Full PTSD	KIDNET (6) WL (6)	16/15	8–14 (11.9)	45.2%	Sri Lanka	UCLA PTSD Index	37.9 (14.2)	2	6
Foa 2013, [42]	Sexual abuse	Full PTSD and Subclinical PTSD	PE (14) ACG (14)	31/30	13–18 (15.3)	100.0%	Germany	CPSS-I	27.3 (7.4)	14	12
Peltonen 2019, [24]	Mixed^a^	Subclinical PTSD	NET (10) TAU (ND)	29/21	9–17 (13.2)	42.0%	Finland	CRIES	38.3 (14.4)	10	3
Rossouw 2018 [43]	Interpersonal trauma	Full PTSD	PE (7–14) ACG (7–14)	31/32	13–18 (15.4)	87.3%	South Africa	CPSS-I	34.5 (8.2)	7–14	6
Ruf 2010, [44]	Mixed^b^	Full PTSD	KIDNET (8) WL (8)	13/13	7–16 (11.5)	46.2%	Multiple^c^	UCLA PTSD Index	43.3 (12.3)	4	6
Schauer 2008, [45]	Mixed^d^	Full PTSD	NET (6) ACG (6)	25/22	11–14 (13.1)	61.7%	Sri Lanka	CAPS-CA	63.2 (17.7)	20	13

*Abbreviations*: *PE = *Prolonged Exposure Therapy, *NET =* Narrative Exposure Therapy, *KIDNET =* Narrative Exposure Therapy for children, *ACG* = Active Treatment Group, *WL* = waiting list, *TAU* = treat as usual, *UCLA PTSD Index* = University of California, Los Angeles Post-Traumatic Stress Disorder Reaction Index, *CPSS-I* = Child PTSD Symptom Scale-Interview, *CRIES =* The Children’s Revised Impact of Events Scale, *CAPS* = Clinician-Administered PTSD Scale, *NA =* Not Available, *w =* week, *m* = month

^a^ Refugeedom (war), Family violence

^b^ Children had experienced an average of four different traumatic event types, mostly prior to leaving their country of origin. Violent attacks against their parents or other family members at home were the most common

^c^ Turkey (Kurdish) /Balkan/ Syria/ Chechnya/ Russia/Georgia/ Germany (Balkan)

^d^According to the classification of researchers, more than 16 types of trauma were included. Such as natural disasters, traffic accidents, sexual assault, etc.

### Primary outcomes

**Fig. 2:**
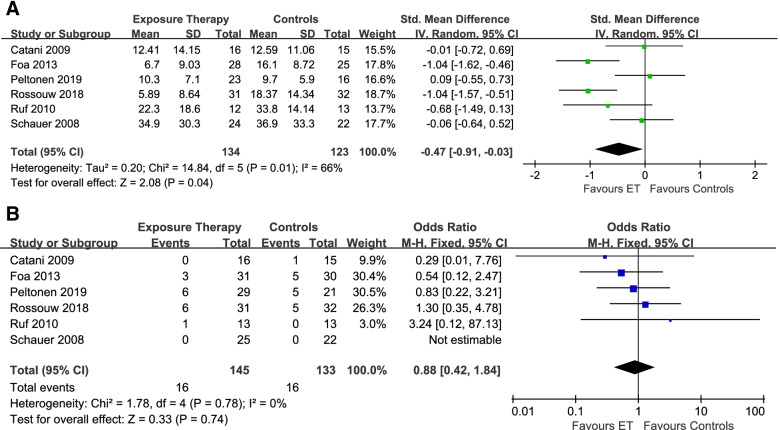
The efficacy (**A**) and acceptability (**B**) of exposure therapy at post-treatment

For evaluating the efficacy of ET in reducing the post-treatment PTSD symptom severity, 6 articles (*n* = 257) with a moderate significant heterogeneity (I^2^ = 66%, *P* = 0.01, Fig. 2A) were included [24, 41, 44, 46, 47]. The results showed that ET was more effective than control groups (SMD: − 0.47, 95%CI: − 0.91 to − 0.03). For acceptability, 6 studies [24, 41, 42, 44, 46, 47] (*n* = 278) with no significant heterogeneity (I^2^ = 0%, *P* = 0.78, Fig. 2B) were included and there were no significant differences between the treatment group and the control group (OR: 0.88, 95%CI: 0.42 to 1.84).

### Secondary outcomes

**Fig. 3:**
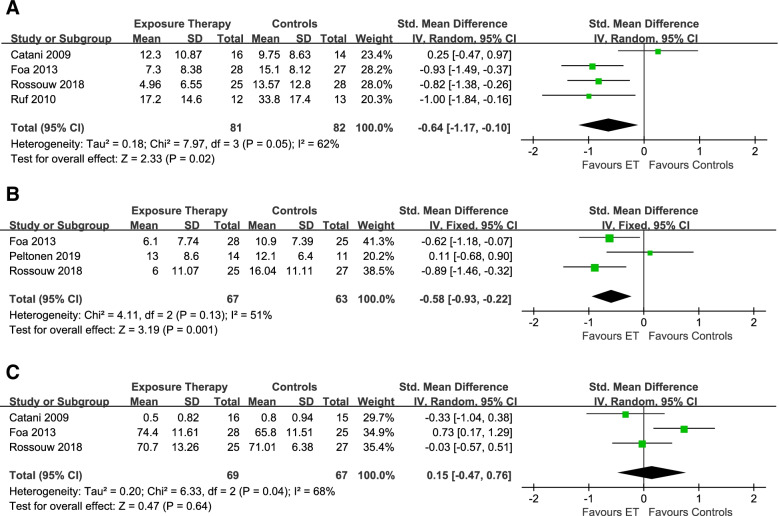
The efficacy of exposure therapy at 1–12 months follow-up (**A**), the effect of exposure therapy on depressive symptoms (**B**), and the effects of exposure therapy on quality of life/social functioning (**C**)

For the effects of follow-up, 4 studies [41, 42, 44, 47] with moderate heterogeneity among studies (I^2^ = 62%, *P* = 0.05, Fig. 3A) were eligible (*n* = 163). Exposure therapy was statistically significantly more efficacious than control conditions (SMD: − 0.64, 95%CI: − 1.17 to − 0.10). For assessing the effects of treatment on depressive symptoms, we included 3 studies [24, 42, 47] (*n* = 130) with non-significant low heterogeneity (I^2^ = 51%, *P* = 0.13, Fig. 3B), and the exposure therapy were more efficacious than control groups (SMD: − 0.58, 95%CI: − 0.93 to − 0.22). In terms of the effects of quality of life/social functioning, 3 trials [41, 42, 47] (*n* = 136) were included with statistically significant moderate heterogeneity (I^2^ = 68%, *P* = 0.04, Fig. 3C). There were no significant differences at the end of treatment (SMD: 0.15, 95%CI: − 0.47 to 0.76).

### Subgroup analysis

**Fig. 4:**
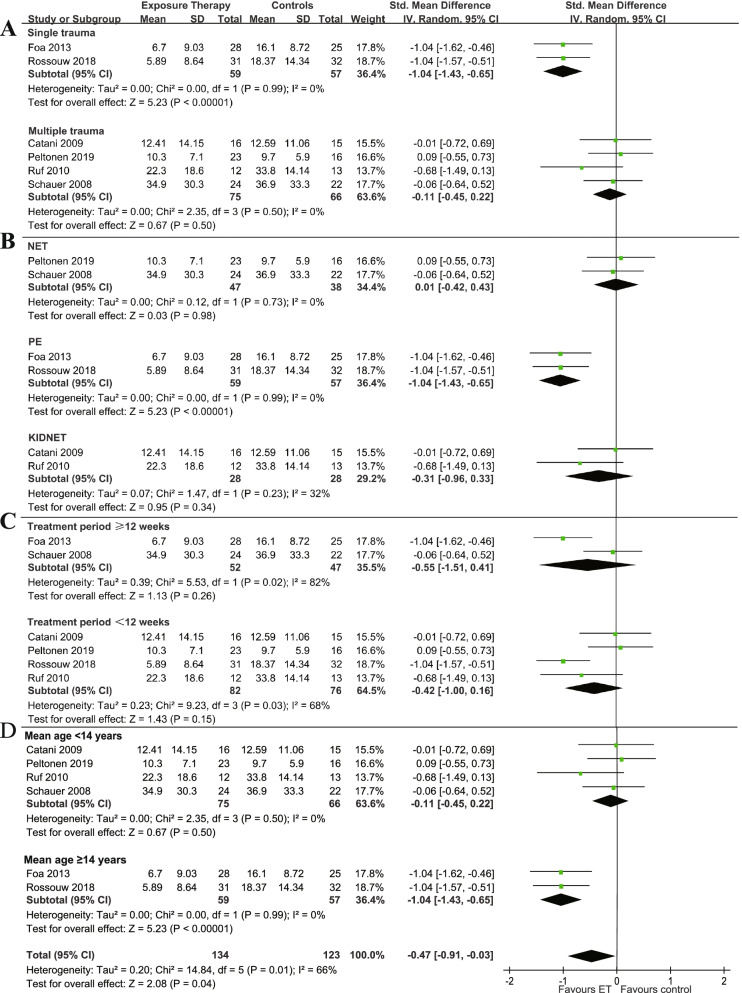
The subgroup analysis of the efficacy at post-treatment

In the subgroup analysis of the post-treatment efficacy among patients who suffered multiple types of trauma, no significant difference was found between exposure therapy and control groups (SMD: − 0.11, 95%CI: − 0.45 to 0.22; I^2^ = 0%, *P* = 0.50, Fig. 4A). For patients who suffered single type of trauma, exposure therapy was statistically more efficacious than control groups (SMD: − 1.04, 95%CI: − 1.43 to − 0.65; I^2^ = 0%, *P* = 0.99). The intervention method using PE as the experimental group (SMD: − 1.04, 95%CI: − 1.43 to − 0.65; I^2^ = 0%, *P* = 0.99, Fig. 4B) was more effective than control groups, but groups using NET (SMD: 0.01, 95%CI: − 0.42 to 0.43; I^2^ = 0%, *P* = 0.73) or KIDNET (SMD: − 0.31, 95%CI: − 0.96 to 0.33; I^2^ = 32%, *P* = 0.23) were not. For patients whose treatment sessions were more than 12 weeks (SMD: − 0.55, 95%CI: − 1.51 to 0.41; I^2^ = 82%; *P* = 0.02, Fig. 4C) or less than 12 weeks (SMD: − 0.42, 95%CI: − 1.00 to 0.16; I^2^ = 68%, *P* = 0.03), exposure therapy was not more effective than control groups. For patients with an average age of less than 14 years, ET did not show a significant difference between ET and the control groups (SMD: − 0.11, 95%CI: − 0.45 to 0.22; I^2^ = 0%, *P* = 0.50, Fig. 4D). For patients with an average age of 14 years and older, ET was more effective than the control groups (SMD: − 1.04, 95%CI: − 1.43 to − 0.65; I^2^ = 0%, P = 0.99).

### Quality assessment

**Fig. 5:**
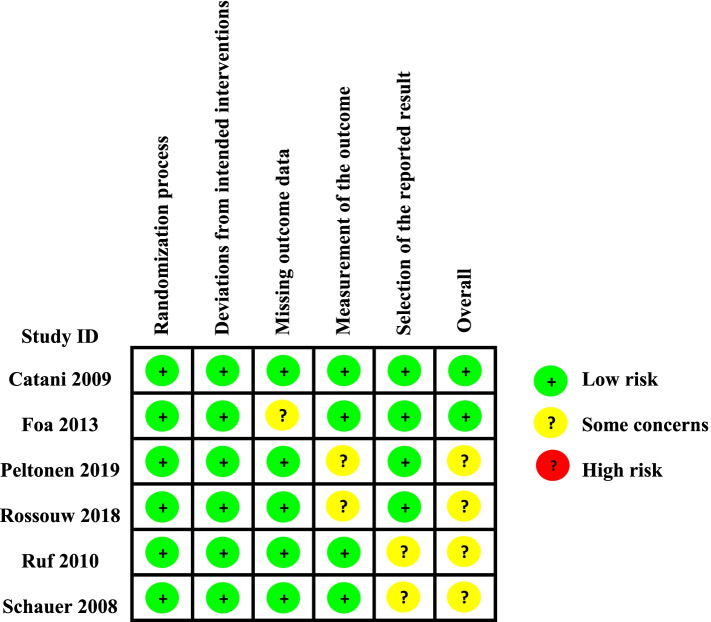
Risk of bias graph: review authors’ judgements about each risk of bias item presented as percentages across all included studies

As assessed with Risk of bias tool 2.0 (ROB 2.0), 2 RCT [37, 41] was rated as low risk, and four RCTs [24, 42, 44, 46, 47]were rated as some concerns (Fig. 5).

## Discussion

This meta-analysis is aimed to assess the efficacy and acceptability of exposure therapy in the treatment of PTSD in children and adolescents. To our knowledge, this is the first meta-analysis to evaluate the efficacy of exposure therapy in children and adolescents with PTSD. We found that exposure therapy showed higher efficacy than control groups at post-treatment/follow-up and depressive symptom, but the acceptability did not perform better. Subgroup analysis showed that patients with single type of trauma may benefit more from exposure therapy. And PE showed a significant advantage over NET and KIDNET. This meta-analysis may provide insights for the clinical treatment of PTSD in children and adolescents.

It has been proved that ET showed a significant advantage in treating adults [40, 48] and has been recommended as the first-line therapy for adults’ PTSD [16, 40]. This study showed a similar result in terms of PTSD in children and adolescents. Owing to the mechanisms of ET, it can activate the traumatic memory and inserted the safe components [49], so that ET has good efficacy of PTSD. Besides, subgroup analysis showed PE had significant advantages in the treatment of PTSD in children and adolescents. The possible reasons could be the following: Firstly, PE is an exposure-based CBT of PTSD and has been in development since 1982 [16]. Therefore, PE has been widely studied with more comprehensive evidence for PTSD. Secondly, PE is an extensively studied form of individual CBT, the adaptation emphasizes developmental sensitivity, modularity, and flexibility [16]. In addition to the core ET components of psychoeducation and exposure, PE includes more extensive case management and relapse prevention component, which could contribute to the benefits of PE [16]. For NET, on the other hand, the fear structure needs to be activated in a safe environment to decrease maladaptive associations [19]. During re-experiencing of the traumatic events (such as through nightmares, intrusive thoughts, or flashbacks), the fear network becomes reinforced because of the additional layer of emotional distress, and the memory is thus more susceptible to being triggered later [19]. Subgroup analysis showed that exposure therapy was more effective for children and adolescents with single type of trauma. However, this conclusion should be treated with caution due to the limited number of studies included. The trauma types of patients in the present study were more comprehensive compared with the previous study [50]. Four out of six studies in our meta-analysis included patients with multiple trauma types. For example, one of our studies [46] included the teenagers who suffered natural disasters, traffic accidents, or sexual assault. These results were consistent with previous studies [51, 52]. Since patients who experienced multiple types of trauma are more susceptible to complex PTSD including (in addition to the core PTSD symptoms of re-experiencing, avoidance, and hyperarousal) disturbances in affect regulation, dissociation, self-concept, interpersonal relationships, somatization, and systems of meaning [53], complex mental illnesses may have a negative impact on the treatment results of PTSD patients [54], leading to unhealed trauma. And uncured trauma is a common cause of refractory depression and obsessive-compulsive disorder [55].

Our analysis suggested that ET may generally result in better outcomes than control conditions in the long-term follow-up. When treating children and adolescents with trauma, it may be important to not only tackle the one event in their traumatic history, but also all the events that may still cause PTSD symptoms. The clinical model of repeated traumatization underlying ET draws on dual representation theories of PTSD and emotional processing theory and the idea of fear networks [56]. ET constructs a narrative that covers the patient’s entire life, while giving a detailed account of past traumatic experiences, which can contribute to the long-term efficacy [16]. For acceptability, no significant difference was observed between ET and control groups. However, from the patient’s perspective, especially for children and adolescents, exposure therapy is challenging and its treatment process can be relatively painful [15]. Traumatic experience is required to reproduce on the patient, which may cause adverse effects. The treatment cycle is also relatively long [16]. In the treatment duration ≥12 weeks and < 12 weeks, the result was not significant difference. At present we didn’t find more relevant literature to make comparison on this issue, we may need more studies on the correlation between different treatment duration and treatment results in the future. What’s more, we found a difference in the effectiveness of interventions for PTSD symptoms in children (< 14 years) and adolescents (≥ 14 years) at post-treatment. The results showed that exposure therapy was not superior than control groups on children, while it was more effective than control groups for adolescents. These results are consistent with the findings of earlier studies [42, 43, 50], where exposure therapy was found to be superior to control groups in terms of PTSD symptom reduction. However, for children, exposure therapy didn’t show better performance than treatment strategies in other control groups. This can be attributed to the often chronic and recurrent nature of PTSD symptoms [57]. Some studies [58, 59] suggests that prior to age 14 teens become more emancipated from adult authorities while identifying more with the emergent norms of their peers, and after age 14 their created identity is internalized. And we think this character changes may correlate with our results.

Because PTSD patients usually have comorbidities, such as depression and anxiety [60, 61], we also considered the efficacy of exposure therapy for PTSD comorbidities. This study showed that exposure therapy can significantly improve patients’ depressive symptoms. Regarding the mechanism of its effect on depression symptoms, ET may change depression through cognitive shifts or exposure-induced emotional arousal [62] despite the lack of Socratic questioning, specific instruction about cognitive errors, and assigned practice. The specific mechanism is still unclear. However, these indicate that ET could be an effective choice for PTSD patient comorbidity with depressive symptoms in children and adolescents. The patient’s quality of life at the end of treatment was not significantly improved. We speculated that this may be related to the relatively painful process of exposure therapy and further studies are needed.

Overall, our results provided some new perspectives on exposure therapy for PTSD in children and adolescents. We have tried to reduce the heterogeneity among studies by omitting RCTs that may cause significant bias in the results due to article characteristics (such as inappropriate study design, non-RCT, sample size less than 10, etc.). However, due to the following limitations, the results of this meta-analysis should be interpreted carefully. First of all, this study included a small sample size. The scope of our literature search was as wide as possible, but after screening papers in strict accordance with our standards, only six studies were included. One of the reasons for the limited number of studies is the high drop-out rate and the limited number of RCTs for children and adolescents with PTSD. As the mental health of children and adolescents is essential to the sustainable development of society, we decided to adhere to this theme. The reasons for the high drop-out rate include: a. the decision of discontinuing the treatment by the patients themselves or their parents because of the feeling that the treatment was no longer needed [24]; b. the difficulty of re-experiencing traumatic events as reported by NET clients [24]; c. the child populations in wars are usually on the move. ‘Home’ is often a Displaced People’s Camp, a Cross border Transit or Refugee Camp. It is challenging to extend individual services, which last several weeks if not months [46]. Another reason for the limited number studies is that psychotherapy for children and adolescents requires systematic and standardized training, which is more difficult than better quantitative evaluation of drug therapy. In addition, exposure therapy can evoke traumatic memories in children and adolescents, which may account for the small number of variables included in this studies. Secondly, most of the analyzed studies presented with moderate to high heterogeneity. These could come from the clinical and methodological characteristics. Some subgroup analyses were conducted to explore the potential sources of heterogeneity, however, other important parameter such as symptom severity, gender, country of patients couldn’t not be addressed due to the limitation of original data. Thirdly, the ROB 2.0 showed most of the risk of bias of the included studies was rated as low risk and some concerns. These mainly came from the deviations from intended interventions, for the blinding. However, it was difficult to conduct with double-blinding in psychotherapies. Fourth, due to the limited number of included studies, no publication bias funnel plot was performed, and potential publication bias cannot be ruled out.

## Conclusion

This meta-analysis found that ET showed superiority in terms of efficacy at post-treatment/follow-up and depressive symptoms improvement in treating PTSD in children and adolescents. Patients with single type of trauma may benefit more from the intervention of ET. ET is more effective in patients aged 14 years or older. Moreover, PE could be a better choice for children and adolescents with PTSD. Further well-defined clinical studies should be conducted to confirm those outcomes.

## Data Availability

The datasets analysed during the current study are available in the Mendeley repository, at 10.17632/d6m4xhwtyw.3.
